# Differences in RANTES and IL-6 levels among chronic rhinosinusitis patients with predominant gram-negative and gram-positive infection

**DOI:** 10.1186/s40463-016-0183-x

**Published:** 2017-01-17

**Authors:** Almoaidbellah Rammal, Marc Tewfik, Simon Rousseau

**Affiliations:** 1 Department of Otolaryngology – Head and Neck Surgery, McGill University, Montreal, Canada; 2 Department of Otolaryngology – Head and Neck Surgery, King Abdul-Aziz University, Jeddah, Saudi Arabia; 3 Departments of Medicine, McGill University, Montreal, Canada; 4 McGill University Health Centre, 1001 Decarie Blvd., Rm D05.5718, Montreal, QC, H4A 3 J1 Canada

**Keywords:** RANTES, IL-6, Chronic rhinosinusitis, Gram positive infection, Gram negative infection

## Abstract

**Background:**

Bacteria are suspected players in the pathogenesis of chronic rhinosinusitis (CRS), yet their exact role is not understood. We investigated the effect of planktonic and biofilm of staphylococcus aureus (SA) and Pseudomonas aeruginosa (PA) on the mucosa of CRS patients with gram-positive and gram-negative infections by measuring the levels of IL-6 and RANTES, a chemokine with activity on eosinophils and T lymphocytes.

**Methods:**

Ethmoid mucosa of six CRS patients with gram-positive bacteria on culture and five with gram-negative bacteria were compared to ethmoid mucosa of 8 control patients. The tissue explants were stimulated with SA and PA extracts in planktonic and biofilm form for 6 hours, then RANTES levels were measured by ELISA.

**Results:**

Compared to the control group, CRS patients with gram-negative predominance demonstrated a significantly higher level of RANTES expression in response to all forms of bacterial stimuli (*P*-value <0.05). Patients with gram-positive predominance showed a higher level of RANTES compere to control group, however, this difference was not significant (*P*-value >0.05).

**Conclusions:**

The mucosa of CRS patients with gram-negative infections has a heightened innate immune response compared to controls and patients with gram-positive infections. It is possible that this response leads to the pathological eosinophilic inflammation seen in CRS.

**Electronic supplementary material:**

The online version of this article (doi:10.1186/s40463-016-0183-x) contains supplementary material, which is available to authorized users.

## Background

Chronic rhinosinusitis (CRS) is defined as an inflammatory condition of the paranasal sinuses and nasal passages that persists for a minimum of 8 to 12 weeks [1, 2]. The prevalence rate among the general Canadian population is estimated to be 5%. There is general agreement that no one causative factor fully explains or adequately accounts for the pathologic manifestations and clinical heterogeneity of rhinosinusitis [3, 4].

CRS can be divided based on the presence of nasal polyps into: CRS with nasal polyp (CRSwNP) and without nasal polyp (CRSsNP). It was thought that the CRSwNP was the endpoint of the CRSsNP, however, this hypothesis is no longer accepted [5]. The two categories are completely different entities based on clinical presentation, distinct inflammatory pathways, cytokine profiles, and different tissue remodeling [5]. T_H2_ lymphocyte is the predominant type of lymphocyte in CRSwNP that results in recruitment of eosinophil, whereas CRSsNP is characterized by higher levels of neutrophil because the predominant type of lymphocyte is T_H1_ lymphocyte.

Bacteria are suspected players in the pathogenesis of chronic rhinosinusitis (CRS), yet their exact role is not understood. Bacterial infection is one of the most important causative factors in CRS. Another study suggests that bacteria and fungi are more likely to be disease modifiers rather than primary causative agents in predisposed individuals [6].

Gram-positive bacteria are the most common cause of infection in CRS patients. Staphylococcus bacteria are the predominant cause type of infection in gram-positive patients; whereas in gram-negative infections, P.aeruginosa is the most common cause of infection. In a retrospective study done by Wormald et al., S.aureus was found to be the most common isolated bacteria in CRS patients (35%), followed by P.aeruginosa (9%), Haemophilus spp (7%) and S. pneumonia (5%) [7].

Bacteria exist in two forms in the sinus - planktonic and biofilm forms. It has been estimated that more the 99% of bacteria exist in biofilm form [8]. A biofilm is defined as “a microbially derived sessile community characterized by cells that are irreversibly attached to a substratum or interface or to each other, are embedded in a matrix of extracellular polymeric substances that they have produced, and exhibit an altered phenotype with respect to growth rate and gene transcription” [9]. A significant number of studies identified biofilms in the mucosa of CRS as ranging from 25% to 92% [8].

Innate immunity is the first line of defense against infection. Unlike adaptive immunity, the innate immune system lacks specificity. However, innate immune effector cells recognize the pathogens through their pathogen-associated molecular patterns (PAMP). PAMPs have several important features relevant to innate immunity; they are found only in the pathogen and never in the host cell, and an entire class of pathogens shares the same PAMPs. For example, gram-negative bacteria all have lipopolysaccharide (LPS) as a cell wall component and gram-positive bacteria all have lipoteichoic acid (LTA) [10].

IL-6 is the key cytokine responsible for the transition from innate immunity to adaptive immunity [11]. In Cantero et al.’s study, IL-6 was higher in S.aureus biofilm treated mucosa compared to the control group [12].

Regulated on Activation, Normal T cell Expressed and Secreted (RANTES) is a member of the CC chemokines family and a well-known major eosinophil attractant [13, 14]. Beck et al. demonstrated the presence of RANTES immunoactivitiy in the nasal polyp biopsies [14]. Teran et al. reported that RANTES is released into the supernatant of the cultured nasal polyp tissue [15]. Immunohistological studies showed that RANTES is present on the nasal epithelium of patients with nasal polyps [14].

Our objective was to investigate the innate immune response of CRS patients with gram-positive and gram-negative infections to planktonic and biofilms of staphylococcus aureus (SA) and Pseudomonas aeruginosa (PA) by measuring the level of RANTES, and IL-6. The working hypothesis of this research was that CRS patients with different types of predominant infections have different innate immune responses to bacteria at the mucosal level. These altered immune responses may explain in part the bacterial species predominance within the sinuses, the severity of the disease phenotype, and the general susceptibility to disease development.

## Methods

### Study design and populations

A case-controlled prospective study was undertaken. Eleven patients with CRS and eight control subjects that had undergone a transsphenoidal resection of a pituitary tumor were included in this study. The research protocol was approved by the McGill University Health Centre ethics committee and informed consent was obtained from all subjects. A Nonparametric (Mann–Whitney) tests with Dunn’s multiple comparisons test were used for the statistical analysis using GraphPad Prism (version 7.0).

Patients with CRS were selected according to the definition of the American clinical practice guidelines for acute and chronic rhinosinusitis [2]. Patients were recruited following inclusion criteria, 1) Documented diagnosis of bilateral CRS, 2) Documented positive cultures for either gram-negative bacteria on at least two separate occasions or gram-positive bacteria on at least two separate occasions, 3) Eighteen to sixty-five years of age, 4) Documented failed medical treatment of CRS, and 5) Patients off antibiotics and systematic/intranasal corticosteroid for at least 1 month prior to surgery. Patients with 1) Documented diagnosis of fungal sinusitis, 2) Documented positive fungal culture, 3) Diagnosed Crohn’s disease, 4) Diagnosed immotile cilia syndrome, 5) Diagnosed cystic fibrosis or 6) Diagnosed immunodeficiency syndrome were excluded from this study.

CRS patients were further divided based on the presence or absence of nasal polyps into CRS with nasal polyps (CRSwNP) and CRS without nasal polyp (CRSsNP); the diagnosis was based on the American clinical practice guidelines for acute and chronic rhinosinusitis [2]. Patients who underwent skull base surgery were used as a control in this study. Preoperative paranasal sinus CT scans were used to access the severity of the disease using the Lund–Mackay scoring system.

### Sample collection

Multiple nasal mucosa biopsies were taken mainly from the ethmoid and sphenoid sinuses. Nasal swabs were taken before harvesting the nasal tissue and before administrating the prophylactic antibiotics. The samples were placed immediately on ice and transferred to the Meakins-Christie laboratories (the Research Institute of the McGill University Health Centre). Specimens were washed three times with ice-cold phosphate-buffered saline (PSB) before being cut into pieces of equal weight. Each biopsy piece was distributed to one of 6 experimental groups in 12-wells tissue culture plates and cultured in 75% Dulbecco’s modified Eagle’s medium/25% Hanks’ buffer solution (100 U/mL penicillin G and 100 μg/mL streptomycin) without serum for 72 h. Every 24 h, the medium was refreshed.

### Sample stimulation

On the third day, biopsy pieces were left unstimulated, as control, or were stimulated for 6 hours in duplicate at 37 °C using planktonic Staphylococcus aureus (PSA), biofilm staphylococcus aureus (BSA), planktonic Pseudomonas aeruginosa (PCF) or biofilm Pseudomonas aeruginosa (BCF). Then the media and the nasal tissue were snap-frozen and stored at −80 °C until further analysis. The duration of stimulations was based on previous study that showed; 6 hours stimulations is sufficient time to get expression for both mRNA and proteins [16].

### Bacterial strain and materials

The Pseudomonas aeruginosa strain used in this study was PACF508. This is a stable mucoid clinical isolate from the sputum of a patient with cystic fibrosis (Hôpital Sainte-Justine, Montréal, Canada) [17]. Regarding the Staphylococcus aureus, the strain was purchased from American Type Culture Collection (ATCC, Manassas, VA).

#### Diffusible material preparation

##### Planktonic S. Aureus and P.aeruginosa

Diffusible material from planktonic S.aureus (PSA) was produced in 4% peptone (Fischer scientific, Pittsburgh, PA). S.aureus was grown in 5 mL of desired media in 12 mL test tubes at 37 °C for 72 h with shaking at 250 RPM. Following this growth, the culture was centrifuged at 2100 X *g* for 20 min to pellet cells. The supernatant was collected and filtered through a 0.22 μM filter (Millipore), aliquoted and stored at −20 °C for use within the month or −80 °C for longer storage. Prior to use, the total protein content was determined via a standard Bradford assay. Similar protocol was used to produce the P. aeruginosa diffusible material.

##### Biofilm S. Aureus and P.aeruginosa

Static PsaDM and S.aureus biofilm were prepared in peptone or SCFM as follows: ~5 × 10^7^ log phase cells were used to seed each 6 mm polystyrene tissue culture wells (Falcon, Franklin Lakes, NJ). After 3 hours of initial attachment, the media was removed, replaced with fresh media, and the attached bacteria were incubated statically at 37 °C for an additional 24 h. After this time, the attached cells were scraped off the plate and were combined with the cell suspension within the well. Bacteria were centrifuged at 2100 X *g* for 30 min and the supernatant was collected and filtered through a 0.22 μm filter. Total protein of filtrates was measured by the Bradford method. Prior to use, bacterial filtrates were heat inactivated at 95 °C for 10 min (to inactivate proteases) and allowed to cool to room temperature.

### Elisa

Human CCL5/RANTES (DY278) DuoSet ELISA kit was purchased from R&D Systems (MN, USA). A human IL-6 ELISA kit (900-K16) was purchased from PeproTech (NJ, USA). All primers were ordered from Invitrogen (Carlsbad, CA.). Berube et.al. [18] 100 μL of supernatant collected after cell stimulation was directly used for RANTES quantification or diluted 1:50 for IL-6 quantification per the manufacturer’s protocol. (Additional files 1 and 2).

## Results

A total of eight control and 11 patients were recruited. Of the eleven, six had predominant gram-positive infections, three of which had CRSwNP, and five had gram-negative infections, three of which had CRSwNP. Baseline characteristics of patients population is shown in Table 1. Four patients had previous sinus surgery, two patients with gram-positive infections and two patients with gram-negative infections. All patients have no allergy.

**Table 1:** Baseline characteristics of patients population

	Control	Case	
		Gram Positive	Gram Negative
No.	8	6	5
Mean Age	58.5	43.5	56.2
Gender (M:F)	4:4	4:2	3:2
Lund–Mackay score	0	13.6	16
CRSwNP		3	3
CRSsNP		3	2

### Rantes

#### Controls vs. patients

**Fig. 1:**
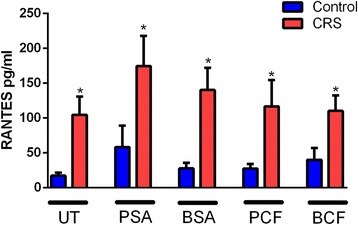
*Comparison of RANTES level between control subjects and CRS patients with different type of bacterial stimulation*. The RANTES levels were significantly higher in CRS patients compared to controls in all type of stimulations. Untreated UT; Planktonic Staphylococcus Aureus PSA; Biofilm Staphylococcus Aureus BSA; Planktonic Pseudomonas aeruginosa PCF; Biofilm Pseudomonas aeruginosa BCF

A strong RANTES production was observed in all CRS patients. The difference between the levels of RANTES secreted by the mucosa in response to all bacterial stimuli, in control and in CRS patients was statistically significant as shown in Fig. 1. Moreover, the unstimulated sample from CRS patients showed a significant amount of RANTES when compared to the unstimulated sample from the control group (*P* = 0.005). Comparison of RANTES production in response to PSA stimuli between control and CRS patients revealed a significantly higher level of RANTES (*P* = 0.02). Similarly, stimulated nasal mucosa taken from CRS patients released a significantly higher level of RANTES compared to stimulated nasal mucosa samples taken from control patients in response to BSA (*P* = 0.02), PCF (*P* = 0.02) and BCF (*P* = 0.02). The highest level of RANTES was seen when the samples from CRS were stimulated by PSA (160.32 ± 39.65).

#### Control vs. gram-positive vs. gram-negative

**Fig. 2:**
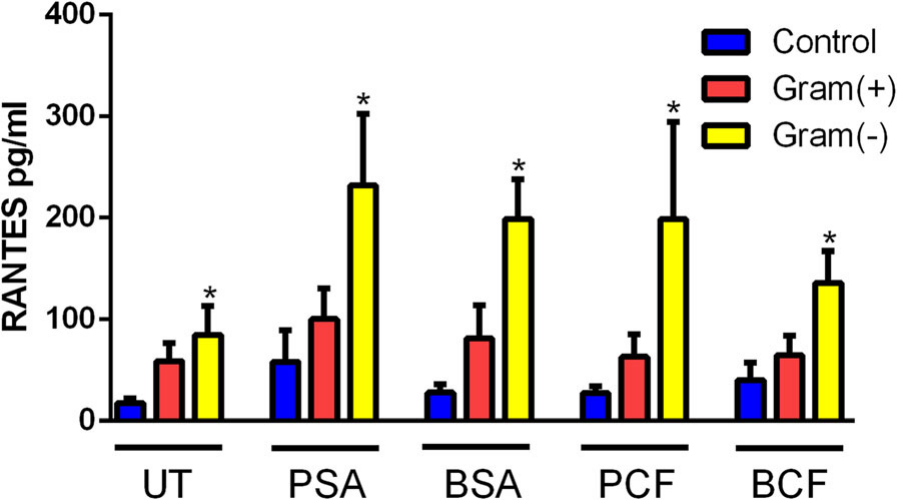
*Difference of RANTES level in CRS with gram-positive and negative infections compared to control group in response to different types of bacterial stimulation.* CRS patients with Gram-negative infection have a significantly higher level of RANTES compared to patients with predominant gram-positive infection and to control subjects. Untreated UT; Planktonic Staphylococcus Aureus PSA; Biofilm Staphylococcus Aureus BSA; Planktonic Pseudomonas aeruginosa PCF; Biofilm Pseudomonas aeruginosa BCF

A subgroup analysis showed that mucosal samples taken from CRS patients with predominant gram-negative infections had a stronger production of RANTES in response to all types of stimuli compared to control and CRS patients with predominant gram-positive infections, (Fig. 2). In the case of unstimulated samples, CRS patients with predominant gram-negative infections produced higher level of RANTES compared to control patients and CRS patients with predominant gram-positive infections and the difference between the control group and gram-negative patients’ group was statistically significant, *P* = 0.01, however, no statically significant difference was observed between gram-positive patients’ when compared to gram-negative patients’ group. The strongest production of RANTES was observed when the mucosal samples taken from CRS patients with predominant gram-negative infections were stimulated with PSA; and when this result was compared to control group, the difference was statistically significant, *P* = 0.03. However, when the RANTES level produced in response to PSA stimulation in CRS patients with predominant gram-negative infections was compared to CRS patients with predominant gram-positive infections, the result was statistically insignificant. Stimulation by BSA also induced RANTES production to a higher level in CRS patients with gram-negative infections compared to controls and to CRS patients with predominant gram-positive infections and the difference was only statistically significant, *P* = 0.0053, when the non-CRS group was compared to CRS patients with predominant gram-negative infections. Moreover, Planktonic form of P.aeruginosa significantly induced higher RANTES production from the mucosal samples harvested from CRS patients with predominant gram-negative infections compared to mucosa from patients with predominant gram-positive and non-CRS patients, with a *P*-value = 0.02. BCF stimulation induced similar results, *i.e.* the highest production of RANTES was observed with gram-negative mucosa followed by gram-positive mucosa and control (*P* = 0.03).

#### CRSwNP vs. CRSsNP

**Fig. 3:**
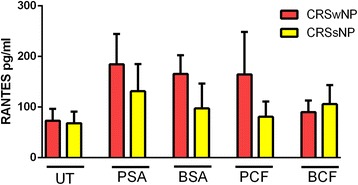
*Difference of RANTES level in CRSwNP and CRSsNP in response to different types of bacterial stimulation.* CRS patients with nasal polyps have a significantly higher level of RANTES compared to patients without nasal polyps. Untreated UT; Planktonic Staphylococcus Aureus PSA; Biofilm Staphylococcus Aureus BSA; Planktonic Pseudomonas aeruginosa PCF; Biofilm Pseudomonas aeruginosa BCF; Chronic Rhinosinusitis with nasal polyps CRSwNP; Chronic Rhinosinusitis without nasal polyps CRSsNP

Although patients with CRSwNP showed a higher level of RANTES production compared to CRSsNP in response to PSA, BSA and PCF, none of those differences were statistically significant (*P* = 0.51, 0.32 and 0.69, respectively). In unstimulated conditions, no statically significant differences were observed between CRSwNP and CRSsNP. Similarly, stimulated mucosa by BCF did not show any significant difference in RANTES production between CRSwNP and CRSsNP, (Fig. 3).

### IL-6

#### Controls vs. patients

**Fig. 4:**
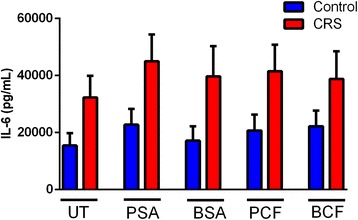
*Comparison of IL-6 level between control subjects and CRS patents with different type of bacterial stimulation*. The IL-6 levels were higher in CRS patients compared to controls in all type of stimulations, however, the difference was statistically insignificant. Untreated UT; Planktonic Staphylococcus Aureus PSA; Biofilm Staphylococcus Aureus BSA; Planktonic Pseudomonas aeruginosa PCF; Biofilm Pseudomonas aeruginosa BCF

No significant differences were observed in IL-6 production from samples taken from CRS patients in response to various stimuli compared to the control group; however, there was a trend for higher IL-6 production among CRS patients. The difference between the production of IL-6 from the unstimulated samples in the case group and the control was statistically insignificant, *P* = 0.14. Additionally, stimulation by PSA did not show a statistically significant difference (*P* = 0.17) in IL-6 production in CRS patients compared to controls. Similarly, no significant differences were observed when the IL-6 levels produced by the control group in response to all types of bacterial stimulation were compared to the CRS group, namely: BSA (*P* = 0.19), PCF (*P* = 0.11) and BCF, (with *P*-value equal 0.19, 0.11 and 0.26 respectively), (Fig. 4).

#### Control vs. gram-positive vs. gram-negative

**Fig. 5:**
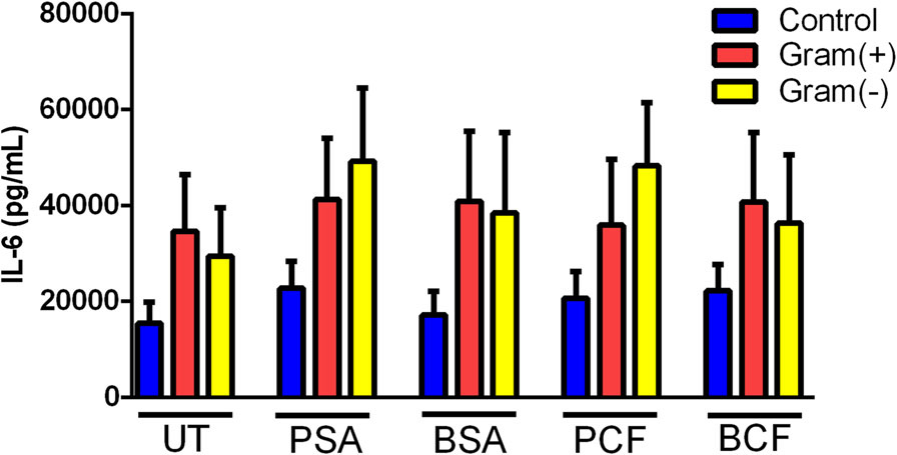
*Difference of IL-6 level in CRS with gram-positive and negative infections compared to control group in response to different types of bacterial stimulation.* CRS patients with Gram-negative infection have no significant difference in IL-6 level compared to patients with predominant gram-positive infection and to control subjects. Untreated UT; Planktonic Staphylococcus Aureus PSA; Biofilm Staphylococcus Aureus BSA; Planktonic Pseudomonas aeruginosa PCF; Biofilm Pseudomonas aeruginosa BCF

In the unstimulated samples, the difference between CRS patients with predominant gram-positive infections compared to control and to CRS patients with predominant gram-negative infections were insignificant. However, the trend observed was of a higher production of IL-6 in patients with gram-positive infections compared to control and to CRS patients with predominant gram-negative infections (Fig. 5). In the case of PSA stimulation, CRS patients with predominant gram-negative infections produced higher levels of IL-6 compared to control patients and to CRS patients with predominant gram-positive infections; the difference between these groups was statistically significant. Stimulation by BSA did not show statistically significant differences in IL-6 production between the three groups. Moreover, BSA induced almost equal amounts of IL-6 production in CRS patients with gram-negative infections and patients with predominant gram-positive infections and controls. No significant difference was observed when IL-6 production following PCF stimulation of mucosa from gram-negative infected patients compared to the gram-positive group and to the control group. Moreover, mucosal stimulation with biofilm form of P.aeruginosa did not show statistically significant difference in IL-6 production; however, higher level of IL-6 was observed in patients with predominant gram-negative infections and in patients with gram-positive infections compared to non-CRS patients, (Fig. 5).

#### CRSwNP vs. CRSsNP

**Fig. 6:**
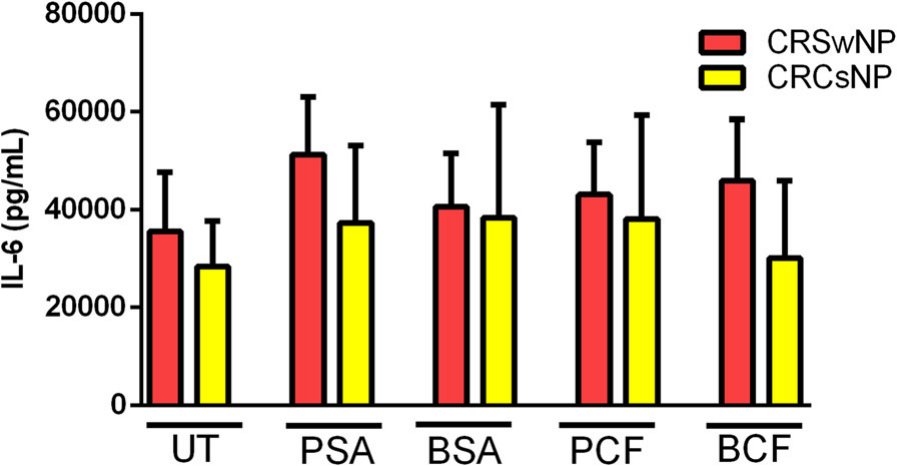
*Difference of IL-6 level in CRSwNP and CRSsNP in response to different types of bacterial stimulation.* CRS patients with nasal polyps have similar level of IL-6 compared to patients without nasal polyps. Untreated UT; Planktonic Staphylococcus Aureus PSA; Biofilm Staphylococcus Aureus BSA; Planktonic Pseudomonas aeruginosa PCF; Biofilm Pseudomonas aeruginosa BCF; Chronic Rhinosinusitis with nasal polyps CRSwNP; Chronic Rhinosinusitis without nasal polyps CRSsNP

When the level of IL-6 production in response to PSA was compared in CRSwNP to the level in CRSsNP the results were statistically insignificant. Similar results were observed in response to BCF stimulation. In unstimulated conditions, no differences were observed between CRSwNP and CRSsNP. Similarly, stimulated mucosa by BSA and PCF did not show any differences in IL-6 production between CRSwNP and CRSsNP, (Fig. 6).

## Discussion

The hypothesis of this study is that CRS patients with different types of predominant infections have different immune responses to bacteria at the mucosal level. The role of bacteria has been given a lot of attention in the medical literature, particularly in regards to their effect on alteration of the host immune system. Recently, an increasing amount of rhinology literature has focused on bacteria not as a primary causative factor of CRS, but rather as a disease modifier. To our knowledge, this is the first study to differentiate CRS based on their predominant bacteria found in the sinuses while investigating relevant innate immune responses.

Eosinophils play an important role in the pathogenesis of CRS [19]. Studies have shown that an increased level of tissue eosinophilia is correlated with the severity of the disease and with the risk of disease recurrence [19]. RANTES is a potent chemoattractant for eosinophils [14]. In vivo studies have proven that RANTES induces symptomatic inflammatory response by causing recruitment of eosinophils [20]. In our study, we successfully demonstrated that nasal mucosa taken from CRS patients were primed to produce RANTES to a higher level compared to non-CRS subjects. Cavallari et al. observed that polyps taken from CRS patients showed a higher level of RANTES gene expression compared to the control group [21]. Lane et al. also detected a higher level of RANTES expression in patients with CRS (CRSwNP and CRSsNP) compared to normal nasal mucosa [22]. However, in the present study we did not measure the level of the tissue eosinophil. Interestingly, a higher level of RANTES production was observed in CRS compared to the control group, even without any stimulation. This could be explained by the fact that there are already chronic inflammation processes in CRS patients and subsequent bacterial stimulation will augment this process and result in increased production of RANTES. Also, patient with predominant gram-positive infections did not show a significant increase in RANTES production compare to patients with predominant gram-positive infections. This could be explained by the fact that gram negative bacteria are particularly virulent, hence they elicit more immune response.

Eosinophils have been associated with host protection from parasitic infections [23, 24]. This type of infection elicits a Th2 immune response which results in IL-5 production, an essential cytokine for eosinophil differentiation [23]. In the present study, we observed a higher level of RANTES production, an essential eosinophilic chemotactic, in CRS patients with predominant gram-negative infections, suggesting that perhaps CRS represents a pathological misrecognition of gram-negative bacteria as parasitic which then results in an abnormal eosinophilic response.

Within the clinical realm of sinus disease, gram-negative bacteria are particularly virulent and often are associated with medically and surgically recalcitrant inflammation. Patients with predominant gram-negative infections are very difficult to treat and have a poor response to sinus surgery. Batcharyya et.al showed that 14.8% of post-ESS patients had persistent gram-negative infections; with P.aeruginosa being the most common isolated bacteria [25]. Similar results were observed in the Nadal et al. study that found that gram-negative infections were present in 27% of cultures (16% are P.aeruginosa). Moreover, patients with prior surgeries had a significantly higher level of gram-negative isolate (30%) compared to patients with no prior ESS (9.5%) [26]. In our study, we were able to show that patients with chronic gram-negative infections had a higher response to bacterial stimulation in the form of production of RANTES compared to control and patients with gram-positive infections. This finding is consistent with the clinical picture of gram-negative patients, who often have very severe disease and require multiple surgeries.

Moreover, this finding suggests that bacterial products were able to stimulate the innate immune responses in patients with chronic gram-negative infections compared to patients with gram-positive infections. However, the exact reason why gram-negative patients behave differently is not understood though we could speculate that patients with chronic gram-negative infections have a more severe underlying inflammatory process compared to gram-positive CRS patients, and that this difference has an effect on the innate immune response and gene expression.

CRS is a heterogonous disease, with a wide variety of disease severity and phenotypes; one accepted way to subcategorize CRS is based on the presence or absence of nasal polyps into CRSwNP and CRSsNP. A study by Zhang et al. suggested that they have different and distinct pathophysiology leading to inflammation [27]. However, in our study we did not find a statistically significant difference in either RANTES or IL-6 production between CRSwNP and CRSsNP patients when the tissue explant was subjected to different types of bacterial stimulation, suggesting that those two cytokines could be equally produced between the two spectrums of the disease.

Biofilms have gained a lot of attention in rhinology literature in the last few decades. Studies estimated the presence of biofilms in the sinus of CRS patients to be between 70% and 80% [28, 29]. Patients with biofilms usually presented with more severe symptoms and worse radiological and endoscopy scores compared to biofilm negative patients [29]. The most common biofilm forming organisms are S.aureus and P.aeruginosa. S.aureus biofilm is associated with severe and recalcitrant CRS [30]. Wormald et al. showed that biofilm forms of S.aureus were able to elicit an intense immune response when the sinonasal tissue explants were stimulated for 24 h compared to unstimulated explants [30]. In our study, we were able demonstrate that the biofilm forms of S.aureus and P.aeruginosa are both able to induce an inflammatory response in gram-negative CRS predominant patients but not in gram-positive predominant patients or in non-CRS patients by producing RANTES, which is a potent eosinophil chemotactic agent. Again the virulent nature of gram-negative bacteria and their ability to recur could have an impact on the innate immune response.

The link between biofilms and chronic diseases is well established. Biofilms are suspected to cause diseases by using several mechanisms: for example, continuous planktonic cell detachment and the release of endotoxin and exotoxin are well-documented [31, 32]. Moreover, biofilms are highly resistant to both host immunity and antibiotics. Studies estimate that biofilms are able to persist despite using 100 to 1000 times the concentrations of antibiotics and biocides that can inhibit planktonic cells [31]. Also, host immunity cells are not able to eliminate biofilms as they would in a planktonic infection [31].

## Limitation

One of the limitations of this study is the sample size; however, we consider this study to be a pilot study, since no similar previous work has been done, especially in subcategorizing CRS based on the culture result. We used a simple culture method to detect the predominant organisms in the sinus. Although one could argue that a more sophisticated method would have detected more organisms, we think that the simple culture is still the most widely used and clinically relevant method in hospital settings.

## Conclusion

Our study demonstrates that CRS patients with predominant gram-negative infections produce a higher level of RANTES in response to different types of bacteria, and it is possible that this response might participate to the influx of eosinophilic inflammation in CRSwNP and CRSsNP patients. This could suggest that the mucosa of CRS patients with gram-negative infections has a heightened innate immune response compared to controls and to patients with gram-positive infections.

## Additional files

Additional file 1: ELISA for RANTES. (DOCX 16.2 kb)
Additional file 2: ELISA for IL-6. (DOCX 13.4 kb)

## Abbreviations

CRS: Chronic Rhinosinusitis
CRSwNP: Chronic Rhinosinusitis with nasal polyp
CRSsNP: Chronic Rhinosinusitis without nasal polyp
RANTES: Regulated on Activation, Normal T cell Expressed and Secreted
IL-6: Interleukin-6
LPS: Lipopolysaccharide
LTA: Lipoteichoic Acid
PAMP: Pathogen-associated molecular patterns
SA: Staphylococcus aureus
PA: Pseudomonas aeruginosa
