# Supplementary material for: Differences in RANTES and IL-6 levels among chronic rhinosinusitis patients with predominant gram-negative and gram-positive infection
Source: J Otolaryngol Head Neck Surg. 2017 Jan 17;46:7. doi: 10.1186/s40463-016-0183-x (PMC5240439; doi:10.1186/s40463-016-0183-x)
Supplement: Additional file 1: — ELISA for RANTES. (DOCX 16.2 kb) [file 40463_2016_183_MOESM1_ESM.docx]

**A****dditional file 1: ELISA for RANTES**

ELISA was performed according to the following protocol: captured antibodies were diluted in PBS to a concentration of 1.0μg/ml for RANTES. Then 100μl was immediately added to each ELISA plate well and the plate was then sealed and incubated overnight at room temperature. The following day, the wells were aspirated and washed four times using 300μl of wash buffer (0.05% tween-20 in PBS). Then 300μl of blocking buffer (1% BSA) was added to each well and incubated at room temperature. One hour later, all wells were again washed four times. Standards were diluted 1:120. Then eight standard curve points were made using 2-fold serial dilution in reagent diluent (1% BSA in PBS) ranging from 1000pg/ml to zero. For the samples, the media were centrifuged for three minutes at 10,000g and the supernatant were collected. Then 100μl of stander and sample was added to each well in the plate and incubated at room temperature for at least two hours. After that, the plates were washed again four times and 100μl of diluted detection antibody in reagent diluent (to a concentration of 20ng/ml (1:120)) was added to each well and incubated at room temperature. Two hours later, the wells were aspirated and washed again four times and 100μl of Streptavidin-HRP (diluted 1:200) was added to each well then the plate was covered and incubated for 20 minute at room temperature. The plates were then washed four times and 100μl of Substrate solution (1:1 mixture of color reagent A (H_2_O_2_) and color reagent B (Tetramethylbenzidine)) (R&D Systems Catalog # DY999) were added to each well and the plate was covered and incubated for 20 minutes at room temperature. Finally, 50μl of Stop solution (2N H_2_SO_4_) was added per well. The plate was then immediately placed in the ELISA plate reader at 450 nm with correction set at 570 nm to determine the optical density of each well. Concentrations of RANTES in media supernatants were calculated from the standard curve. Levels of RANTES are expressed in picograms per milligram of tissue.
