# Supplementary material for: Differences in RANTES and IL-6 levels among chronic rhinosinusitis patients with predominant gram-negative and gram-positive infection
Source: J Otolaryngol Head Neck Surg. 2017 Jan 17;46:7. doi: 10.1186/s40463-016-0183-x (PMC5240439; doi:10.1186/s40463-016-0183-x)
Supplement: Additional file 2: — ELISA for IL-6. (DOCX 13.4 kb) [file 40463_2016_183_MOESM2_ESM.docx]

**A****dditional file 2: ELISA for IL-6**

ELISA for IL-6 was performed according to the manufacture protocol (Appendix-II): captured antibodies were diluted in PBS to a concentration of 1.0μg/ml. Then 100μl were immediately added to each ELISA plate well and the plate was then sealed and incubated overnight at room temperature. The following day, the wells were aspirated and washed four times using 300μl of wash buffer (0.05% tween-20 in PBS). Then 300μl of blocking buffer (1% BSA) were added to each well and incubated at room temperature. One hour later, all wells were again washed again four times. Standards were diluted from 1.5ng/ml to zero, 1ng/ml to zero or from 2ng/ml to zero for IL-6 and IL-8, respectively. For the samples, the media were centrifuged for three minutes and the supernatant was collected and diluted 1:50 in diluent (0.05% tween-20 with 0.1% BSA). Then 100μl of standard and sample was added to each well in the plate and incubated at room temperature for at least two hours. After that, the plates were washed again four times and 100μl of diluted detection antibody (to a concentration of 0.5μg/ml) was added to each well and incubated at room temperature. Two hours later, the wells were aspirated and washed again four times and 100μl of Avidin Peroxidase (diluted 1:2000) was added to each well and incubated for 30 minutes at room temperature. The plates then were then washed four times for the last time and 100μl of ABTS liquid substrate solution was added to each well. The plate was then placed in an ELISA plate reader at 405 nm with a correction set at 650 nm to monitor color development. A standard curve was generated for each set of samples assayed.
